# Characterization of DNA Processing Protein A (DprA) of the Radiation-Resistant Bacterium Deinococcus radiodurans

**DOI:** 10.1128/spectrum.03470-22

**Published:** 2022-12-01

**Authors:** Dhirendra Kumar Sharma, Hari S. Misra, Ishu Soni, Yogendra S. Rajpurohit

**Affiliations:** a Molecular Biology Division, Bhabha Atomic Research Centre, Mumbai, Maharashtra, India; b Life Science, Homi Bhabha National Institute (DAE-Deemed University), Mumbai, Maharashtra, India; Ohio State University

**Keywords:** DprA, eDNA, natural transformation systems

## Abstract

Environmental DNA uptake by certain bacteria and its integration into their genome create genetic diversity and new phenotypes. DNA processing protein A (DprA) is part of a multiprotein complex and facilitates the natural transformation (NT) phenotype in most bacteria. Deinococcus radiodurans, an extremely radioresistant bacterium, is efficient in NT, and its genome encodes nearly all of the components of the natural competence complex. Here, we have characterized the DprA protein of this bacterium (DrDprA) for the known characteristics of DprA proteins of other bacteria and the mechanisms underlying the DNA-RecA interaction. DrDprA has three domains. *In vitro* studies showed that purified recombinant DrDprA binds to both single-strand DNA (ssDNA) and double-strand DNA (dsDNA) and is able to protect ssDNA from nucleolytic degradation. DrDprA showed a strong interaction with DrRecA and facilitated RecA-catalyzed functions *in vivo*. Mutational studies identified DrDprA amino acid residues crucial for oligomerization, the interaction with DrRecA, and DNA binding. Furthermore, we showed that both oligomerization and DNA binding properties of DrDprA are integral to its support of the DrRecA-catalyzed strand exchange reaction (SER) *in vitro*. Together, these data suggested that DrDprA is largely structurally conserved with other DprA homologs but shows some unique structure-function features like the existence of an additional C-terminal Drosophila melanogaster Miasto-like protein 1 (DML1) domain, equal affinities for ssDNA and dsDNA, and the collective roles of oligomerization and DNA binding properties in supporting DrRecA functions.

**IMPORTANCE** Bacteria can take up extracellular DNA (eDNA) by natural transformation (NT). Many bacteria, including Deinococcus radiodurans, have constitutive competence systems and can take up eDNA throughout their growth phase. DprA (DNA processing protein A) is a transformation-specific recombination mediator protein (RMP) that plays a role in bacterial NT, and the absence of this gene significantly reduces the transformation efficiencies of both chromosomal and plasmid DNA. NT helps bacteria survive under adverse conditions and contributes to genetic diversity in bacteria. The present work describes the characterization of DprA from D. radiodurans and will add to the existing knowledge of DprA biology.

## INTRODUCTION

Natural competence is a genetically regulated mode of horizontal gene transfer to acquire the extracellular DNA (eDNA) through transformation. Bacteria proficient in natural transformation (NT), acquire external DNA from the environment and recombine it with their genetic material to introduce genetic diversity (1–5) and fitness under adverse environmental conditions through different modes, including using DNA as foodstuffs (6, 7) or utilizing extracellular DNA to facilitate recombination repair of damaged DNA (1, 5, 8). Mechanistically, it involves two major steps: (i) the uptake of external DNA into the cytosol by a macromolecular complex and (ii) the integration of transforming DNA into the host chromosome by homologous recombination (if transforming DNA is chromosomal DNA) or the stabilization of transforming DNA into functional circular plasmid DNA by single-strand annealing (SSA) activity (if transforming DNA is plasmid DNA) (9). The DNA uptake system comprises either type II secretion systems or type IV pili (T4P) (6, 10, 11). The components of the uptake complex have been identified, and their roles in DNA uptake have been characterized. For instance, external double-strand DNA (dsDNA) translocated into the cytoplasm is first converted into single-strand DNA (ssDNA) by the EndA nuclease and then processed by the combined action of the receptor protein ComEA (permease), the transmembrane channel protein ComEC, and the ATPase motor protein ComF (7, 12–14). The internalized ssDNA is protected by single-stranded DNA binding (SSB) protein and by a natural transformation-specific recombination mediator protein (RMP) (DNA processing protein A [DprA]) (15–17). The DprA protein further facilitates RecA recombinase loading onto incoming ssDNA by overcoming the SSB protein barrier (16, 18–20). Finally, transforming DNA is integrated into host genetic material through homologous recombination (6, 9, 21). DprA has been found in almost all bacteria, including Deinococcus radiodurans, and functionally characterized from few bacteria. It is also named CilB or Smf in different bacteria.

D. radiodurans is best characterized by its extraordinary resistance to DNA-damaging agents, including radiation and desiccation (22, 23). *D. radiodurans* in exponential growth phase form diads/tertrads cell morphology and has multiple copies of a multipartite genome system (22–24). The possible translocation of an undamaged copy of a genome element or its constituents from one cell of the tetrad to other cells and the pumping out of damaged material from the cells have been hypothesized for the efficient double-strand break (DSB) repair and radioresistance of this bacterium (24, 25). The molecular basis for transporting such materials across the cells and from the outside environment is not well understood. However, this bacterium exhibits NT and has acquired nearly 10% of its genetic material from other organisms, and, thus, it has created genetic diversity (26, 27). Therefore, the possibility of NT contributing to its extreme phenotypes, albeit indirectly, cannot be ruled out and would be worth investigating. The D. radiodurans genome encodes components of the NT system, including DprA (DrDprA). It has been shown that the RecFOR complex/DdrB protein partially compensates for the functional requirement for DrDprA in the DNA transformation process (28). Determining the molecular basis of DrDprA that makes it different from the DprA proteins of other bacteria would require detailed studies on its structure and functions. Here, we report the structure-function characterization of DrDprA, a core component of the NT system. We demonstrate that DrDprA exhibits almost equal preferences for binding to both ssDNA and dsDNA. The conserved residues for oligomerization and RecA interactions have been identified and confirmed through mutagenesis. DrDprA could support RecA functions and reduce SSB interference in the DNA strand exchange activity of DrRecA. It was able to protect DNA from nuclease degradation, and its deletion mutant was found to be defective in at least plasmid transformation. Taken together, the results of the present study suggest that DrDprA has all of the DNA metabolic properties that are known for other bacterial DprA proteins, along with some unique features, and are required for the transformation of external DNA in this bacterium.

## RESULTS

### DrDprA has conserved structural domains of bacterial DprAs.

**FIG 1:**
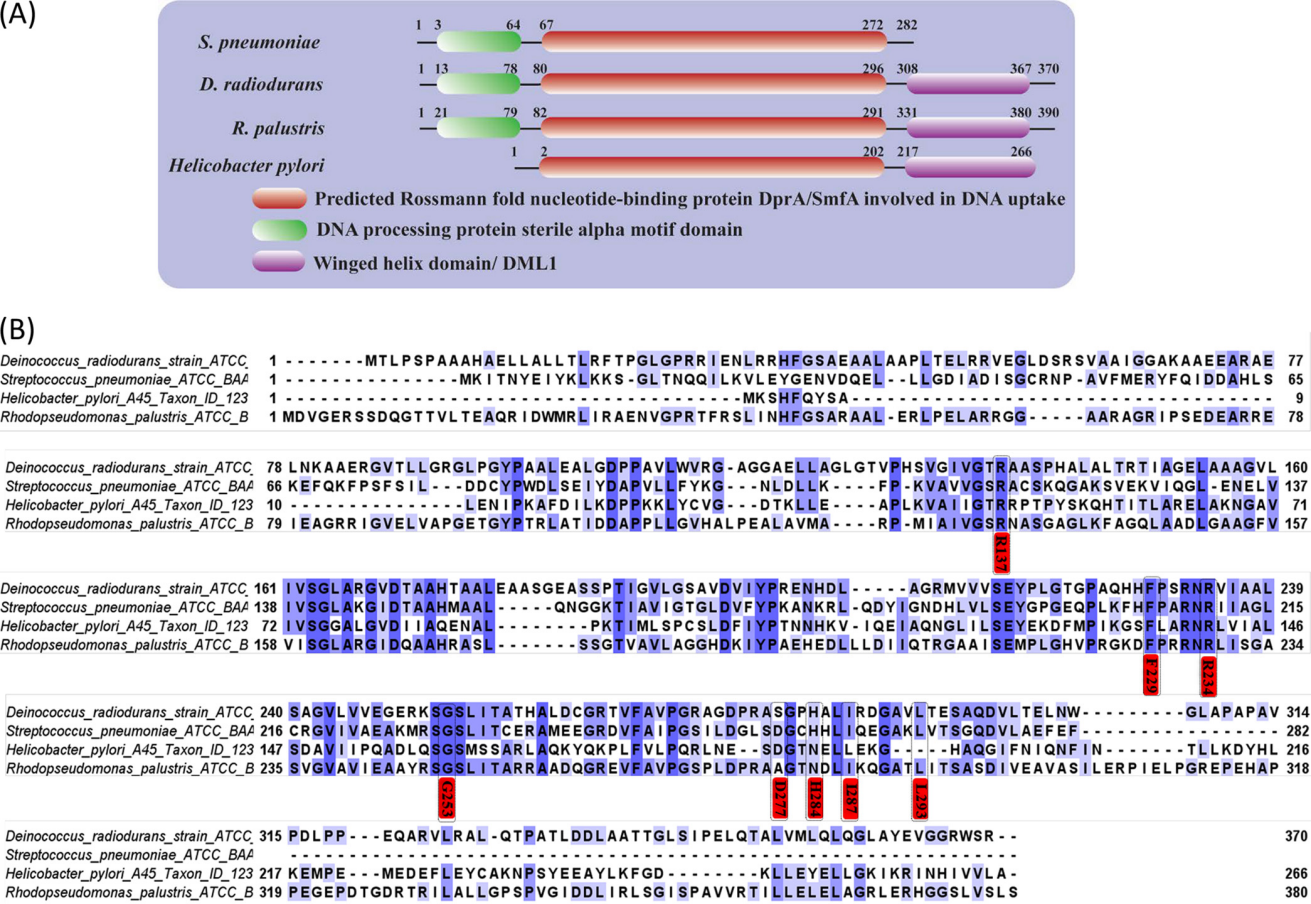
Domain architecture of DprA. (A) Domain architecture of DprA proteins of select bacteria (S. pneumoniae, R. palustris, D. radiodurans, and H. pylori). The central Rossmann fold (RF) is conserved and present in all DprA proteins, while the N-terminal SAM domain is common in all DprA proteins except that of H. pylori. An additional C-terminal DML1 domain is present in the DprA proteins of D. radiodurans and R. palustris. (B) Multiple-sequence alignment of DprA proteins. Red labels represent conserved putative amino acids responsible for the DprA-RecA interaction (I287, G253, and D277), the DprA-DprA interaction (L293, and H284), and the interaction with DNA (R137, R234, and F229), as predicted by *in silico* analysis.

The number of amino acids of the DprA proteins of different bacteria varies from 240 (Campylobacter jejuni) to 398 (*Synechocystis* and Neisseria gonorrhoeae) (29). The DprA protein of D. radiodurans (DrDprA) is 370 amino acids (aa) long (26). The DprA proteins of different organisms show significant similarities at the amino acid sequence level. The multiple-sequence alignment (MSA) of DrDprA with the DprA proteins of Rhodopseudomonas palustris (RpDprA) Helicobacter pylori (HpDprA), and Streptococcus pneumoniae (SpDprA) showed 31 to 38% identity (Fig. 1). Structural studies of the DprA proteins of R. palustris (PDB accession number 3MAJ), S. pneumoniae (PDB accession number 3UQZ) (19), and H. pylori (PDB accession number 4LJK) (30) have revealed that the protein is structurally divided into a central Rossmann fold (RF) domain, an N-terminal sterile alpha motif (SAM) domain, and a C-terminal Drosophila melanogaster Miasto-like protein 1 (DML1) domain. MSA showed that DrDprA contains all three domains; the SAM domain spans amino acids 13 to 78, the RF domain spans amino acids 80 to 296, and the DML1 domain spans amino acids 308 to 367 (Fig. 1). The analysis of the DprA domain diversity among DprA homologs suggested that the SAM domain is generally associated with the RF domain in most DprA proteins except for those of H. pylori and Pyrococcus furiosus, while the DML1 domain is found in the DprAs of Mycobacterium tuberculosis, D. radiodurans, Vibrio cholerae, R. palustris, Neisseria meningitidis (NmDprA), Haemophilus influenzae, and *Synechocystis* sp. (Fig. 1A). The N-terminal SAM and RF domains contribute to dimerization and RecA-DNA interactions, while the C-terminal DML1 domain contributes to the formation of higher-order oligomers (18, 19). In eukaryotes, SAM domain-containing proteins have been shown to regulate several developmental changes (31), while the DML1 domain is considered a Z-DNA binding domain (32).

### DrDprA forms equal-affinity nucleoprotein complexes with ssDNA and dsDNA.

**FIG 2:**
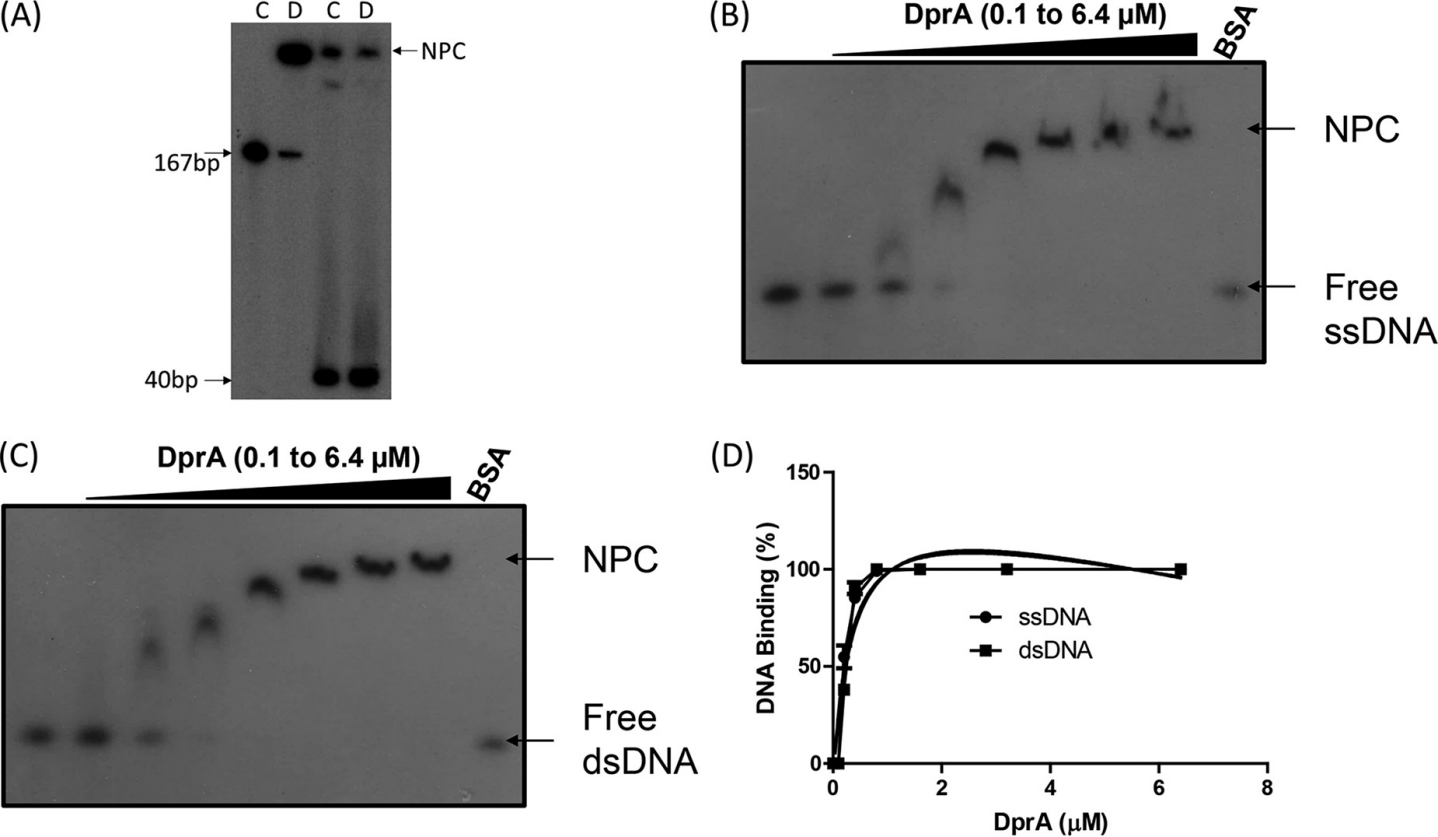
DNA binding activity of DrDprA. (A) The required DNA length for DrDprA binding was measured by incubation with ^32^P-labeled single-strand DNA of two sizes (40-mer and 167-mer). Lane C represents the respective ssDNA without DrDprA, and lane D represents the ssDNA bound with 2 μM DrDprA. (B and C) The 167-mer ssDNA/dsDNA with increasing DrDprA concentrations (0.1, 0.2, 0.4, 0.8, 1.6, 3.2, and 6.4 μM) in a reaction buffer as described in Materials and Methods. Reaction mixtures were separated on 8% native PAGE gels, the gels were dried, and an autoradiogram was done. (D) Both free DNA and DNA bound to protein were quantified densitometrically, and the bound fractions (percentages) were calculated as described in Materials and Methods. The results are plotted as a function of the percentage of bound DNA and the DrDprA concentration (micromolar) using GraphPad Prism software.

**FIG 3:**
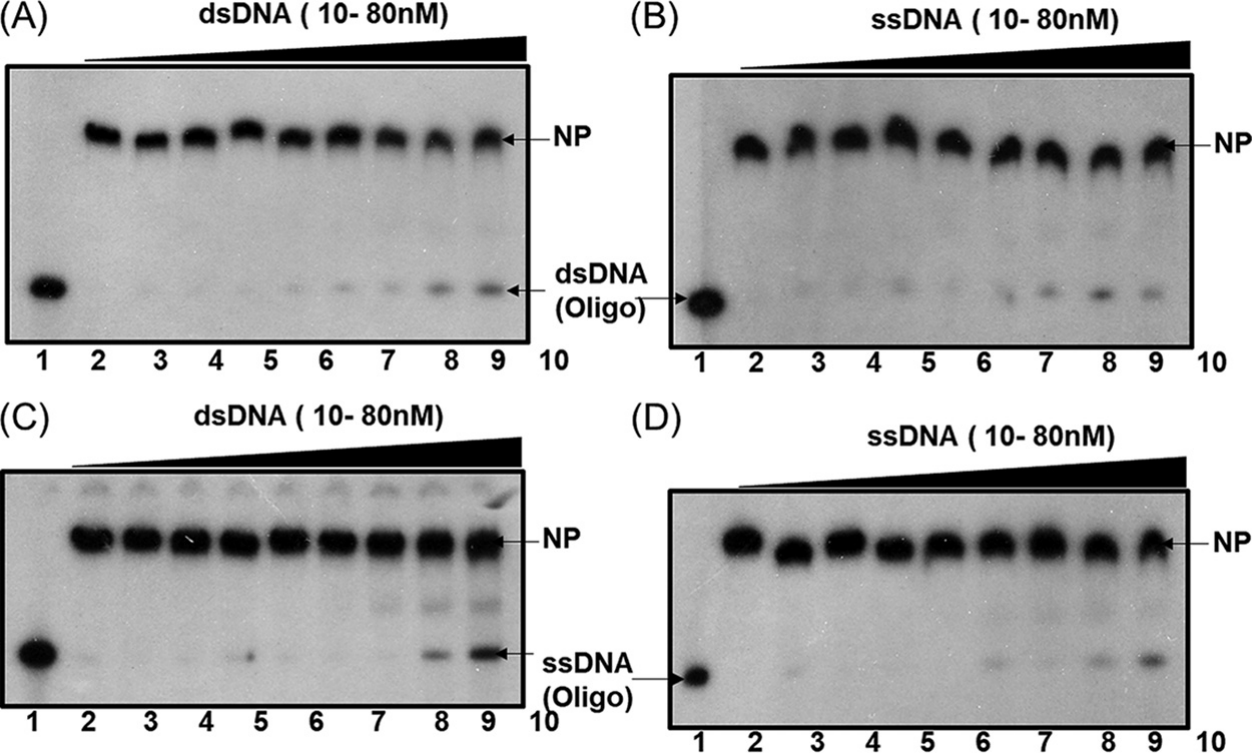
DrDprA DNA binding preference between ssDNA and dsDNA substrates. A total of 1.6 μM purified recombinant DrDprA protein was incubated with ^32^P-labeled dsDNA (A and B) and ssDNA (C and D). Proteins bound to these substrates were chased with increasing concentrations of unlabeled ssDNA (B and D) and dsDNA (A and C). Mixtures were analyzed on a native PAGE gel, and autoradiograms from a reproducible representative experiment are shown. Both free DNA and DNA bound to protein were quantified densitometrically from three independent experiments, and the bound fractions (percentages) were calculated. NP, nucleoprotein.

The recombinant DrDprA protein was purified to near homogeneity (see Fig. S1 at https://barc.gov.in/publications/mbio/dna_mp/spectrum-03470-22.pdf), and the DNA binding activity was monitored for both dsDNA and ssDNA. Since the DNA binding ability of DprA homologs has been shown to increase with increasing lengths of DNA up to 80 bp (20, 33), the DNA length requirement of DrDprA for its binding was evaluated using 40-bp- and 167-bp-long ssDNA substrates. It was observed that DprA forms an unstable NPC (nucleoprotein complex) with DNA of <40 bp and a stable NPC with 167-bp ssDNA/dsDNA (Fig. 2A). Interestingly, DrDprA could bind to both ssDNA and dsDNA (167 bp) with equal affinities. The dissociation constant (*K_d_*) value for binding to ssDNA was 3.039 ± 0.09724 μM, and that for binding to dsDNA was 3.526 ± 0.1172 μM (Fig. 2B to D). Competition experiments were performed to further ascertain the binding preference of DrDprA for ssDNA/dsDNA substrates. The results showed that DrDprA has the same affinities for binding to ssDNA and dsDNA, as the NPCs of both radiolabeled ssDNA and dsDNA could not be destabilized by a 50- to 400-fold molar excess of cold ssDNA and/or dsDNA and vice versa (Fig. 3). This characteristic of DrDprA is unique compared with the DprAs of other bacteria. For example, SpDprA and the DprA protein of Bacillus subtilis (BsDprA) show binding to ssDNA only (17, 19, 20), while HpDprA interacts with both ssDNA and dsDNA, and it modestly prefers ssDNA over dsDNA (34). DrDprA also showed interactions with circular plasmid DNA (M13mp18) incubated with and without ATP (1 mM), indicating that DrDprA does not require DNA ends to bind with DNA (see Fig. S2 at the URL mentioned above). Similar observations of DprA binding to DNA independent of open ends have been reported for SpDprA and HpDprA (20, 34).

The binding of deinococcal proteins that otherwise prefer dsDNA to ssDNA compared with their homologs in other bacteria has been observed previously. A notable example is the preference of DrRecA for dsDNA (35–37). Together, these results suggested that the binding of DrDprA to ssDNA and dsDNA is sequence and DNA end independent. The ability of DrDprA to bind to dsDNA is an interesting phenotype and suggests the possible role of this protein in DNA metabolism beyond natural transformation. The salt and temperature stability of the DrDprA NPC with ssDNA and dsDNA was evaluated, and it was found that at NaCl concentrations of up to 250 mM, more than 90% of the NPC was retained (data not shown). DrDprA or its NPC with ssDNA was found to be stable at temperatures of up to 45°C. Beyond this temperature, the NPC dissociates rapidly irrespective of the DNA binding order and incubation at different temperatures (see Fig. S3 at the URL mentioned above).

### DrDprA interacts with DrRecA.

**FIG 4:**
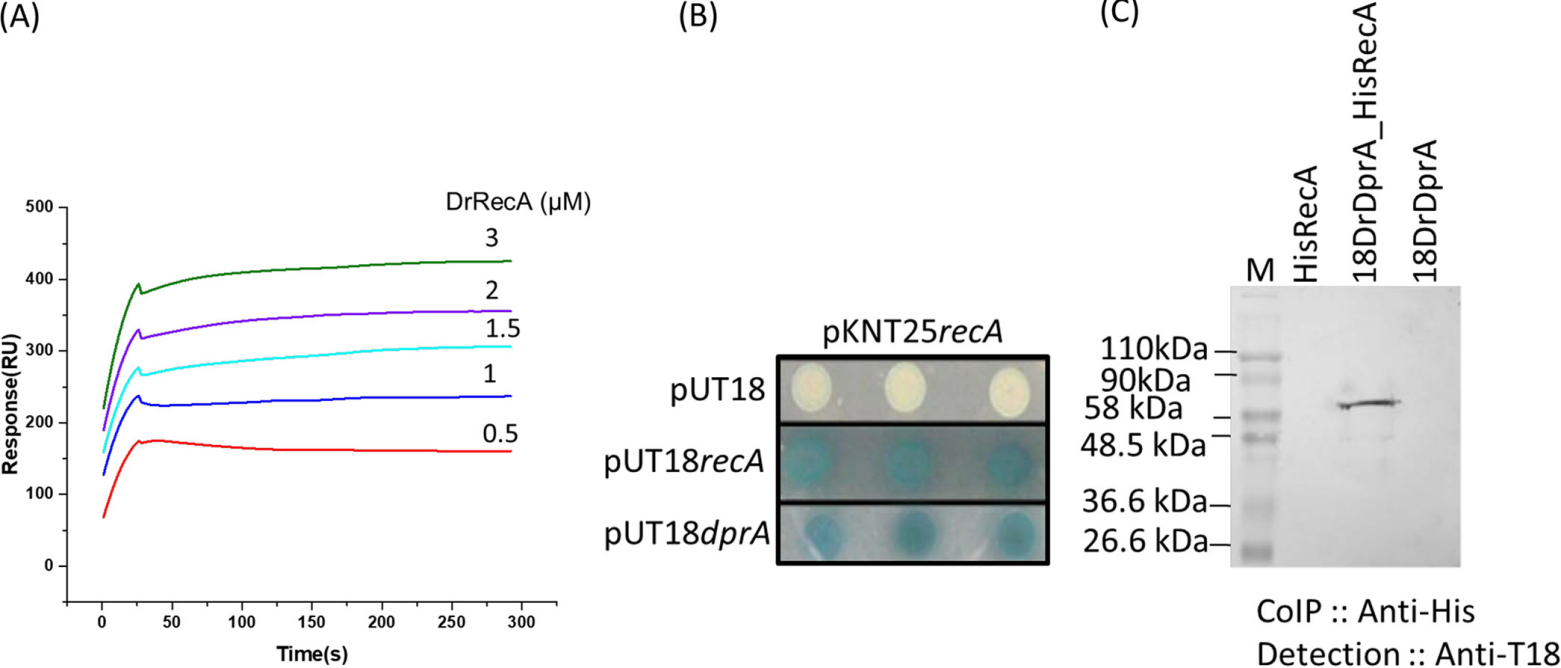
DrRecA-DrDprA interaction in surrogate E. coli cells (*ex vivo*), in D. radiodurans cells (*in vivo*), and *in vitro*. (A) A total of 2.5 μM DrDprA protein was immobilized on a gold sensor chip, followed by incubation with different concentrations (0.5 to 3 μM) of DrRecA. Surface plasmon resonance (SPR) signals were recorded, and data were processed as described in Materials and Methods. The data given are representative of data from experiments repeated three times. RU, response units. (B) T18 and T25 tags of adenylate cyclase alone and with the *dprA* and *recA* genes of D. radiodurans were cloned into BACTH plasmids. These plasmids were transformed into the E. coli BTH101 host. The interactions of proteins tagged with T18 and T25 were monitored as white-blue colonies. RecA-C18 and RecA-C25 were used as positive controls, while C18 and C25 tag-expressing cells were used as negative controls. (C) Cell extracts of D. radiodurans cells coexpressing C18-DrDprA and His-DrRecA from plasmids pVHSM and pBAD, respectively, used for immunoprecipitation assays. Immunoprecipitation was done using anti-His antibody, and immunoprecipitates were separated on an SDS-PAGE gel, followed by immunoblot detection using antibodies against the T18 domain of CyaA (anti-T18 immunoblot), as detailed in Materials and Methods. M, molecular marker.

The roles of some DprAs in the loading of RecA onto ssDNA and their function as a recombination mediator protein (RMP) have been reported previously. Furthermore, the interaction of DprA with RecA has also been shown (17, 18, 20, 38, 39). Therefore, the interaction of DrDprA with the DrRecA protein was monitored *in vitro*, *ex vivo*, and *in vivo*. *In vitro*, the DrRecA-DrDprA interaction was monitored with purified proteins by surface plasmon resonance (SPR) as described in Materials and Methods. The results showed a concentration-dependent increase in the SPR signal when purified DrDprA was brought into contact with DrRecA in solution (Fig. 4A). A strong interaction was supported with a suitable Lorentz fit and a *K_d_* value of 2.93 × 10^−7^ ± 1.34 × 10^−8^ M for the interaction of DrDprA with DrRecA. The *ex vivo* interaction of these proteins was checked in the surrogate Escherichia coli strain BTH101 using a bacterial two-hybrid (BACTH) system. For this, T18-tagged DrDprA and T25-tagged DrRecA were coexpressed in E. coli BTH101 (Fig. 4B). The reconstitution of active adenylate cyclase (CyaA) by joining the T18 and T25 domains of CyaA upon the interaction of these two proteins was monitored by the expression of the β-galactosidase enzyme (31). As expected, cells coexpressing T18-DrDprA and T25-DrRecA produced intense blue colonies on an X-Gal (5-bromo-4-chloro-3-indolyl-β-d-galactopyranoside) plate, which was not observed in cells coharboring T18- and T25-expressing vectors as negative controls (Fig. 4B). The level of expression of β-galactosidase activity was very high, as predicted from the blue color intensity of the bacterial colonies and the activity measured in solution, suggesting a strong interaction between DrRecA and DrDprA *ex vivo* (Fig. 4B). The interaction of DrDprA and DrRecA was also monitored *in vivo*. For this, DrRecA tagged with His_6_ and DrDprA tagged with the T18 domain of CyaA were expressed in D. radiodurans. The prospective interaction was monitored by coimmunoprecipitation (co-IP) using a histidine antibody, and the partner was detected with a T18 antibody. The results showed that lanes representing samples from either DrDprA or DrRecA did not show a signal with the T18 antibody (Fig. 4C). At the same time, those of cells coexpressing His-DrRecA and T18-DrDprA produced a band with a molecular weight of ~56 kDa, which is theoretically the size of T18-DrDprA (Fig. 4C). Together, these results suggested that the DrDprA protein interacts with DrRecA physically.

### The DNA binding and oligomerization abilities of DrDprA are required for its support of RecA functions.

**FIG 5:**
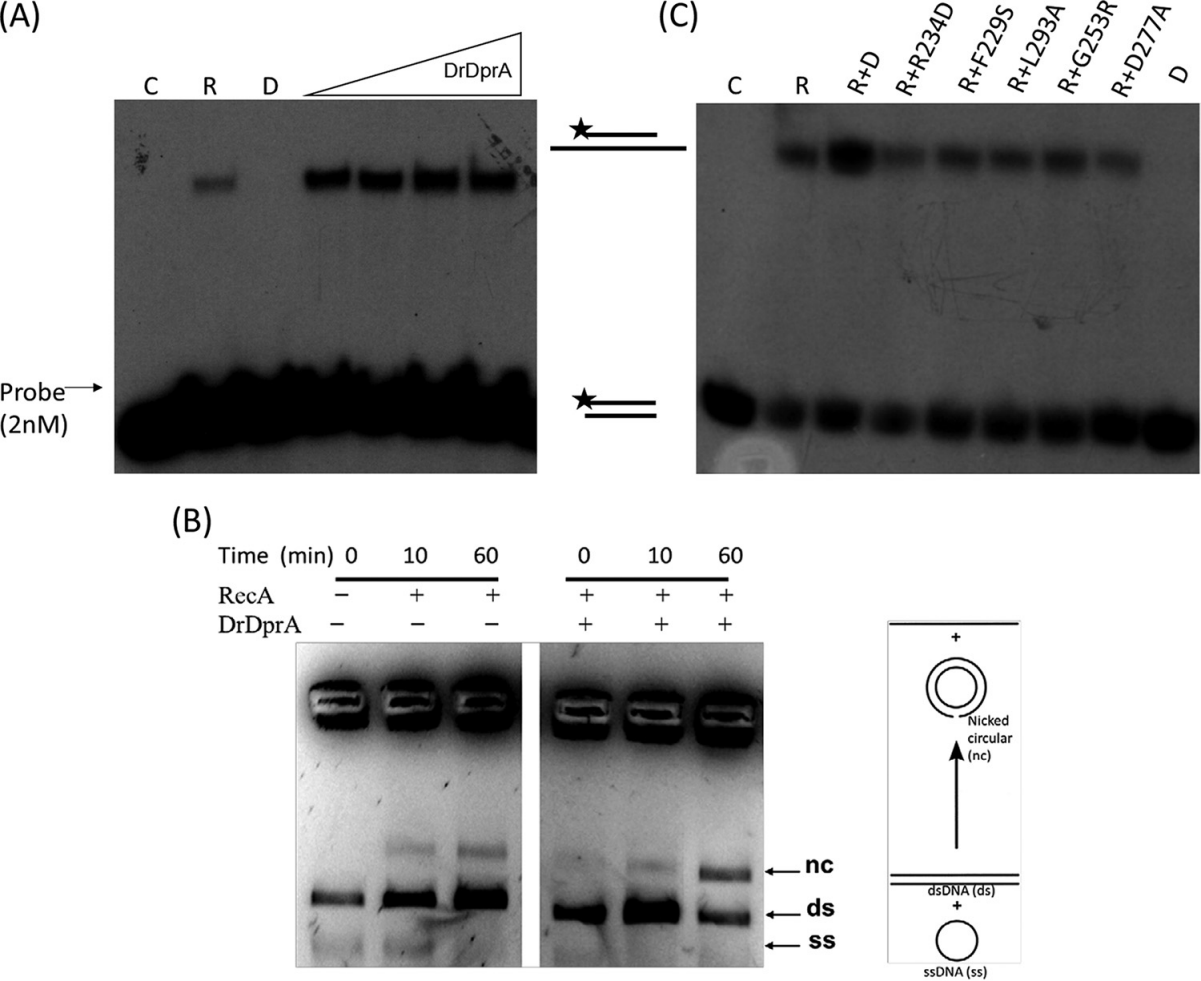
Stimulatory effect of DrDprA on DrRecA-catalyzed DNA strand exchange activity. (A) For oligonucleotide-based DNA strand exchange activity, 0.5 μM DrRecA protein was used. Increasing concentrations of DrDprA (0.5, 1, 2, and 4 μM) were used to observe the stimulatory effect of DrDprA. (B) M13mp18-based DNA strand exchange reaction carried out as detailed in Materials and Methods. The DrRecA and DrDprA concentrations used are 2.5 μM and 2 μM, respectively. (C) Wild-type DrDprA and its different mutant proteins were tested for their stimulatory effect on DrRecA-catalyzed oligonucleotide-based DNA strand exchange activity. C, control DNA; R, DrRecA; D, DrDprA.

DprAs of other bacteria are known to improve the DNA strand exchange reaction (SER) of RecA (18, 20). DrDprA showed a strong physical interaction with DrRecA (Fig. 4). Therefore, the support of the DNA strand exchange activity of DrRecA by DrDprA was evaluated using oligonucleotide-based (short-homology) and M13mp18 DNA-based (extended-homology) DNA strand exchange reactions. A low efficiency of the SER was observed at 0.5 μM DrRecA protein in the oligonucleotide-based reaction, which was significantly improved upon the addition of 0.5 to 4 μM DrDprA (Fig. 5A). Similarly, the addition of 2 μM DrDprA substantially improved the M13mp18 DNA-based SER by DrRecA (Fig. 5B). The improvement of the SER in the presence of DrDprA is in agreement with the similar effects of DprA on recombination reactions in other bacteria. These results suggest that the presence of DrDprA positively impacts the SER by DrRecA.

DrDprA is a DNA binding protein that undergoes oligomerization in solution and is able to interact with DrRecA. Therefore, the involvement of these attributes in the stimulation of the SER of DrRecA was studied. Previously, key amino acids involved in the protein-protein interaction between DprA and RecA were identified (15, 19, 20). Based on these reports, the amino acid sequence of DrDprA was mapped with the DprA proteins of S. pneumoniae, R. palustris, and H. pylori, and the corresponding amino acids residue of DrDprA were mapped. The putative amino acids responsible for the DprA-RecA interaction (I287, G253, and D277), the DprA-DprA interaction (L293 and H284), and the interaction with DNA (R137, R234, and F229) were predicted *in silico* (Fig. 1B). Site-directed mutants of these sites were generated, and the recombinant proteins were purified (see Fig. S1E at https://barc.gov.in/publications/mbio/dna_mp/spectrum-03470-22.pdf). The quality, oligomerization status, and conformational stability of the mutant proteins were ascertained by size exclusion chromatography (SEC), dynamic light scattering (DLS) analysis, and circular dichroism (CD) spectroscopy (see Fig. S4 to S6 at the URL mentioned above). The SEC profiles presented suggested that different mutants have different oligomerization levels (see Fig. S4 at the URL mentioned above). The DprA-DprA interaction (L293 and H284) and the DprA-DNA interaction (R137, R234, and F229) mutants have a higher oligomerization state than wild-type DrDprA, and their elution time is reduced considerably compared to that of wild-type DrDprA (see Fig. S4 at the URL mentioned above). Thus, these data suggested that the wild type and the RecA-DprA interaction mutants (I287, G253, and D277) have similar oligomerization levels, while DprA self-interaction and DprA-DNA interaction mutants have higher oligomerization states (see Fig. S4 at the URL mentioned above).

DLS analysis of these mutants revealed that the wild type and the RecA-DprA interaction mutants (I287, G253, and D277) have average particle diameters in the 20- to 30-nm range, while DprA-DprA interaction (L293 and H284) and DNA binding (R137, R234, and F229) mutants have particle diameters in the 40- to 60-nm range (see Fig. S4 at the URL mentioned above). Thus, both the size exclusion chromatography and DLS data corroborate the oligomerization state of these mutants and suggest that DprA-DprA interaction and DNA interaction point mutants have an impact on their self-interactions and stability in solution. Furthermore, CD spectra were recorded for all mutants, and the data suggested that the changes in the overall secondary structures were minimal, arguing in favor of the lack of a change in the conformational stability of the mutants compared to the wild type (see Fig. S6 at the URL mentioned above).

**FIG 6:**
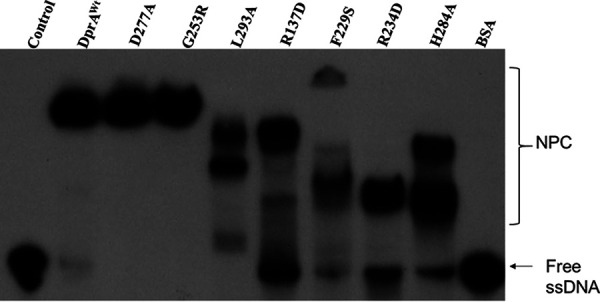
DNA binding activity of DrDprA and its mutant. Recombinant wild-type DrDprA (DprA^Wt^) and its different mutant proteins (DprA-RecA interaction, G253R and D277A; DprA-DprA interaction, L293A and H284A; interaction with DNA, R137D, R234D, and F229S) were purified and incubated with ^32^P-labeled 167-mer single-strand DNA in a reaction buffer as described in Materials and Methods. Reaction mixtures were separated on an 8% native PAGE gel, followed by drying of the gels, and autoradiograms were developed. Experiments were repeated three times and were reproducible.

The functional properties of DrDprA mutants were checked for their corresponding functions *in vitro*. As predicted, the R137, R234, and F229 mutants lost DNA binding activity with a 167-bp-long substrate and also showed altered oligomerization profiles upon size exclusion chromatography compared to the wild type, while predicted oligomer mutants (L293 and H284) showed reduced DNA interactions compared to wild-type DrDprA and defects in oligomerization (Fig. 6; see also Fig. S4 and S5 at the URL mentioned above). The DprA-RecA interaction mutants (G253 and D277) are proficient in DNA interactions similarly to wild-type DrDprA and maintain the wild-type DrDprA profile of oligomerization (Fig. 6; see also Fig. S4 and S5 at the URL mentioned above). When these mutants were tested for their support of the SER function of DrRecA, the stimulatory effect of G253R and D277A (RecA-DprA interaction mutants), L293A (oligomer mutant), and R234D and F229S (DNA binding mutants) on the SER of DrRecA was completely abolished (Fig. 5C). This suggested that in addition to the DrDprA-DrRecA interaction, the oligomerization and DNA binding properties of DrDprA are also crucial for its recombination mediator protein function with DrRecA.

### DrDprA protects DNA from nucleases and limits the inhibitory effect of SSB during the SER.

**FIG 7:**
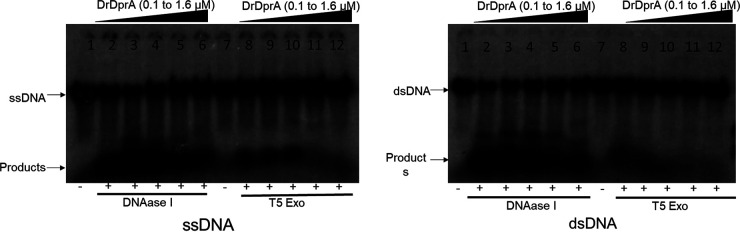
Nuclease protection assay. ^32^P-labeled ssDNA or dsDNA (0.2 nM) either alone or prebound with increasing concentrations of DrDprA (0.1, 0.2, 0.4, 0.8, and 1.6 μM [lanes 2 to 6 and lanes 8 to 12]) was incubated for 30 min with 1 U of DNase I/T5 Exo. Lanes 1 and 7, DNA alone without DrDprA and DNase I/T5 Exo.

DprA binds to transforming DNA and protects it from nucleolytic degradation. The stability and half-life of incoming DNA are reduced in *dprA* mutants (12, 40, 41). Therefore, DNA protection from nucleases was checked in the presence of different concentrations of purified DrDprA. DrDprA (0.1 to 1.6 μM) was able to protect the DNA in the DrDprA-DNA complex from DNase I and T5 exonuclease (Exo) degradation (Fig. 7). This property of DrDprA was found to be similar to that of HpDprA. *In vitro* treatment of the HpDprA-ssDNA complex with ExoT, ExoIII, RecJ, T7 Exo, mung bean endonuclease, and DNase I did not show degradation of complexed DNA (34).

**FIG 8:**
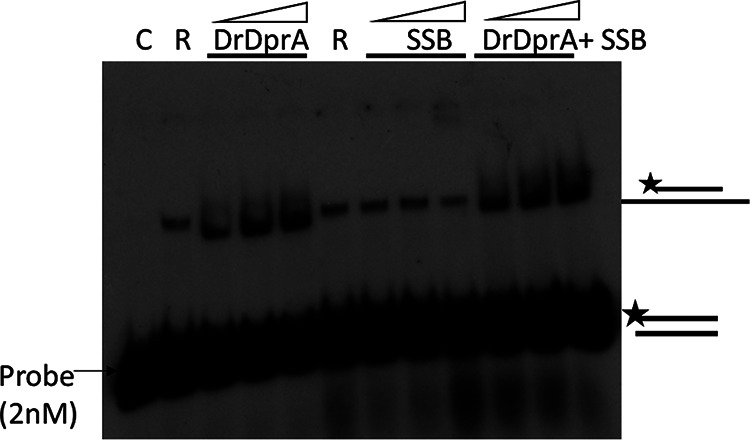
Role of DrDprA in limiting the inhibitory effect of SSB (E. coli) during DNA strand exchange. The DrDprA concentrations used were 1, 2, and 4 μM, while the SSB concentrations used were 0.3, 0.6, and 1.2 μM. C, buffer control reaction; R, DrRecA. The results shown are representative of data from reproducible experiments repeated three times.

It has been shown that the SsbA and SsbB proteins of B. subtilis bind and melt secondary structures in ssDNA but limit RecA nucleation on DNA. DprA of B. subtilis physically interacts with RecA to facilitate RecA nucleation and filament extension on SsbB/SsbB-SsbA-coated ssDNA by dislodging SSB proteins and, thus, assists in RecA-mediated DNA strand exchange in the presence of both SSB proteins (17, 38). Here, we tested the ability of DrDprA to facilitate the DNA strand exchange reaction catalyzed by DrRecA in the presence of E. coli SSB. E. coli SSB has an overall protein architecture and an ssDNA binding surface similar to those of the B. subtilis SsbB protein (42). Preincubation of ssDNA with SSB (0.3 to 1.2 μM) inhibits the DrRecA-catalyzed SER. However, when the SSB-ssDNA complex was incubated with 1 to 4 μM DrDprA followed by the addition of RecA and homologous dsDNA, the DNA strand exchange product yield was improved substantially (Fig. 8), indicating the strong possibility that DrDprA counterbalances the inhibitory effect of SSB on SERs. Thus, the functions of DrDprA are similar to the functions of the BsDprA and RecO proteins in the displacement of SsbB/SsbB-SsbA proteins from ssDNA. It was reported previously that once the action of the DprA/RecO protein gives RecA access to ssDNA, RecA nucleoprotein filament elongation displaces SSB and enables RecA-mediated DNA strand exchange (17).

## DISCUSSION

Bacteria can take up extracellular DNA (eDNA) by natural transformation (NT) through a well-programmed multistep process coordinated by the multiprotein complex (6, 9). Many bacteria, such as D. radiodurans, H. pylori, and N. gonorrhoeae, have constitutive competence systems and can take up eDNA throughout their growth phase (27, 28, 33, 34). DprA is a transformation-specific recombination mediator protein (RMP) that plays a very significant role in NT in the majority of bacteria (17, 18, 19, 20, and 43), and the absence of this gene significantly reduces the efficiency of transformation of both chromosomal and plasmid DNA (28, 44–46). NT helps bacteria survive under adverse conditions and contributes to the genetic diversity of bacteria. D. radiodurans, a radioresistant bacterium, shows natural competence (22, 27, 28), which has been attributed to its genetic diversity, and therefore, the possible role of natural competence in radioresistance cannot be ruled out. A *dprA* deletion mutant of D. radiodurans showed substantial losses and 160- and 21-fold reductions in the frequencies of transformation of chromosomal and plasmid DNA, respectively (28). The effects of the deletion of *dprA* on radioresistance and DSB repair have been reported previously (28). However, our observations differ from their findings and need more studies to conclude. Therefore, the functional characterization of DrDprA would be important for understanding the physiological role of this protein in D. radiodurans. Here, we have found evidence to suggest that DrDprA is largely similar to the DprA proteins of most other bacteria, except that it shows nearly equal affinities for both ssDNA and dsDNA. DprA proteins of many bacterial species have shown a higher affinity for ssDNA than for dsDNA, except for HpDprA, which has shown a moderately higher affinity for ssDNA than for dsDNA (34). Previous structure-function studies of HpDprA and SpDprA identified the potential residues crucial for self-interaction and interactions with DNA and the RecA protein (19, 34). Full-length HpDprA and its RF domain can dimerize *in vitro*, suggesting that the residues involved in dimerization are located at the N-terminal domain (47). However, SpDprA forms tail-to-tail dimers and requires the DML1 domain, and this type of dimerization is crucial for nucleoprotein complexing with ssDNA *in vitro* (19). Sequence alignment studies by Wang et al. identified two motifs in DprA family proteins with essential roles in ssDNA binding (30). Motif 1 (G-S/T/A-R) is located in the loop (b3-a3) region, and the corresponding residues are Gly50 to Arg52 in HpDprA. Motif 2 (F/L/Y-X-X-R-N/D) is located in helix α6 and corresponds to residues Phe140 to Asn144 in HpDprA, and these two motifs are considered to be signatures of the DprA domain that binds to ssDNA (30). MSA analysis of DrDprA with SpDprA, HpDprA, and RpDprA revealed conserved residues for the DprA-RecA interaction (I287, G253, and D277), the DprA-DprA interaction (L293 and H284), and the interaction with DNA (R137, R234, and F229). The amino acid residues Arg137 (R137) and Arg232 (R232) of DrDprA are located in DNA binding motifs 1 and 2, respectively, as identified previously by Wang et al. (30). Amino acid residues contributing to oligomerization and interactions with DNA and DrRecA were found to be largely conserved across DprAs. DrDprA undergoes oligomerization and interacts with DrRecA. Furthermore, DrDprA supports the DNA strand exchange reaction of DrRecA. RecA-DprA interactions have been shown for many bacterial species (S. pneumoniae, R. palustris, and B. subtilis) but not for H. pylori (17, 18, 20, 38, 39). RecA specifically interacts with DprA, which helps with the loading of RecA onto SSB-coated ssDNA (44). The DNA binding activity and oligomerization functions of DrDprA were essential for its support of the SER by DrRecA. The perturbation of the functional DrDprA-DrRecA interaction with oligomer and DNA binding mutants of DrDprA is not surprising. A similar observation was reported previously in the case of SpDprA and its cognate RecA (19). Furthermore, it has been shown that DrDprA dislodges SSB bound to ssDNA and thus removes the inhibitory effect of SSB on the SER by RecA (48). DrDprA binds with ssDNA and protects it from nucleolytic degradation. The DNA protection ability of the DprA protein was also reported previously for SpDprA, NmDprA, and BsDprA (20, 33, 38). Together, our results of the biochemical characterization of DrDprA suggested that, like other DprA proteins, DrDprA has functional conservation of all typical DprA properties. Yet it has some dissimilar properties; for instance, it has all three domains (SMF, RF, and DML1) instead of the two domains known for most DprA proteins except NmDprA and RpDprA. On a functional basis, DrDprA has strong dsDNA binding properties along with ssDNA binding. These dsDNA binding properties suggest the more diverse function of DrDprA in DNA metabolism, including natural transformation.

## MATERIALS AND METHODS

### Bacterial strains, growth medium, and plasmids.

The wild-type bacterium D. radiodurans R1 (ATCC 13939) and its mutants were grown in tryptone-glucose-yeast extract (TGY) medium (1% Bacto tryptone, 0.1% glucose, 0.5% yeast extract) with the appropriate antibiotic, as described previously (49). The E. coli NovaBlue strain was used for the cloning and maintenance of plasmids. E. coli BTH101 (lacking *cyaA* [referred to here as BTH101]) was used to perform *in vivo* protein-protein interaction studies by coexpressing the cloned gene on bacterial two-hybrid (BACTH) plasmids and was grown at 30°C (50). Recombinant proteins were purified from overexpressing E. coli Top10 cells. The pUT18, pKNT25, and pBAD plasmids and their derivatives were maintained in E. coli cells (NovaBlue) in the presence of the required antibiotics. Various molecular biology techniques and their working protocols were used, as described previously (51). The antibody against the T18 (catalog number SC-13582) domain of the CyaA protein of Bordetella pertussis was procured from Santa Cruz Biotechnology, Inc., and the anti-His antibody was purchased from New England BioLabs (NEB). Molecular-biology-grade enzymes, chemicals, and salts were procured mainly from Sigma Chemical Company, Roche Biochemicals (Mannheim, Germany), New England BioLabs, and Merck India Pvt. Ltd. ^32^P-radiolabeled nucleotides were supplied by the Board of Radiation and Isotope Technology (BRIT), Department of Atomic Energy, India. The bacterial strains, plasmids, and primers used in the current study are listed in Table S1 at https://barc.gov.in/publications/mbio/dna_mp/spectrum-03470-22.pdf.

### Recombinant plasmid construction and protein purification.

Table S1 at https://barc.gov.in/publications/mbio/dna_mp/spectrum-03470-22.pdf shows a list of plasmids and primers used in this study. DrDprA with a translational fusion of a T18 tag at the C terminus, referred to as T18-DrDprA, is encoded by plasmid pUT*dprA*. Plasmids pRadHisRecA, pUT18recA, and pKNT25recA were constructed previously and transformed into E. coli BTH101 cells along with plasmid pUT*dprA* (52). The expression of fusion proteins in BTH101 cells was confirmed by Western blotting using antibodies specific for the C18 tag and the polyhistidine tag. T18-tagged DrDprA was cloned into the pVHSM shuttle vector at the NdeI-XhoI sites for coimmunoprecipitation studies in D. radiodurans. Both pVHSM*dprA* and pRadHisRecA were cotransformed into D. radiodurans cells, and coimmunoprecipitation studies were done as mentioned below. Recombinant DrDprA and its various mutants were overexpressed in E. coli Top10 cells using 0.2% (vol/vol) arabinose. Proteins were purified as described previously (35, 53). In brief, E. coli Top10 cells expressing recombinant proteins were harvested 3 h after induction with arabinose. The cell pellet was suspended in buffer A (20 mM Tris-HCl [pH 7.6], 300 mM NaCl, 10% glycerol) containing 0.5 mg/mL lysozyme, 1 mM phenylmethylsulfonyl fluoride (PMSF), 0.03% NP-40, 0.03% Triton X-100, and 10% glycerol and incubated at 37°C for 30 min. Protease inhibitor pellets (NEB) were added to the reaction mixture, and lysis of the cells was done by sonication for 10 min using 5-s pulses with intermittent cooling for 10 s at 35% amplitude. The cell lysate was centrifuged at 12,000 rpm for 30 min at 4°C. A NiCl_2_-charged fast-flow-chelating Sepharose column (GE Healthcare) was equilibrated with buffer A (20 mM Tris-HCl [pH 7.6], 300 mM NaCl, 10% glycerol), and a clear cell extract was loaded onto it. The column was washed with 20 column volumes of buffer A containing 20 mM imidazole until detectable proteins stopped coming from the column. Recombinant column-bound protein was eluted with buffer A containing 250 mM imidazole. Fractions were analyzed on an SDS-PAGE gel, and those containing nearly pure proteins were pooled. Recombinant proteins were further purified on Q-Sepharose, MonoQ, and Superdex-200 columns. Protein fractions free from detectable nuclease contamination and with >95% purity were pooled and precipitated by ammonium sulfate precipitation, followed by dialysis in buffer B (10 mM Tris-HCl [pH 7.6], 50 mM KCl, 50% glycerol, and 1 mM PMSF) and storage at −20°C.

### Protein-protein interaction studies, Western blotting, and coimmunoprecipitation.

As detailed previously, *ex vivo* protein-protein interaction studies were done using a BACTH system (54, 55). E. coli BTH101 cells were transformed with plasmids pUT18recA, pUT18*dprA*, and pKNT25recA expressing the target proteins with a T18 tag or a T25 tag at the C terminus. Empty vectors (pUT and pKNT) in BTH101 cells were used as controls. The cells were spotted onto LB agar plates containing 5-bromo-4-chloro-3-indolyl-β-d-galactopyranoside (X-Gal) (40 μg/mL), isopropyl-β-d-thiogalactopyranoside (IPTG) (0.5 mM), and antibiotics as required. The plates were incubated at 30°C for 12 h, and the appearance of white-blue colonies was recorded. For the Western blot and coimmunoprecipitation studies, plasmids pVHSM*dprA* and pRadHisRecA expressing C18-tagged (C18-DrDprA) and His-tagged (His-RecA) fusion proteins were cotransformed into D. radiodurans. The recombinant cells coexpressing these proteins were induced with 0.5 mM IPTG, and the harvested cells were washed with 70% ethanol, followed by lysis in buffer (50 mM Tris base, 150 mM NaCl, 5 mM EDTA) containing 0.5% Triton X-100, 1 mM PMSF, 1 mM dithiothreitol (DTT), 0.5 mg/mL lysozyme, and 50 μg of a protease inhibitor cocktail tablet. After sonication, clear cell extracts were obtained by centrifugation at 12,000 × *g* for 30 min. The clear cell extracts were used for immunoprecipitation using polyclonal antibodies against the His tag, the immunoprecipitates were separated on a 10% SDS-PAGE gel, and protein was transferred to a polyvinylidene difluoride (PVDF) membrane and hybridized with monoclonal antibodies specific for the T18 tag. Anti-mouse secondary antibodies conjugated with alkaline phosphatase were used to detect the color signal formed by the BCIP (5-bromo-4-chloro-3-indolyl phosphate)-NBT (nitroblue tetrazolium) substrate (Roche Biochemical, Mannheim, Germany).

Surface plasmon resonance (SPR) (Esprit; Autolab, Netherlands) was used to study the interaction of DrDprA with DrRecA. A total of 2.5 μM DrDprA protein was immobilized on a bare gold sensor chip by employing N-ethyl-N’-(3-(dimethylamino)propyl)carbodiimide/N-hydroxysuccinimide (EDC-NHS) chemistry according to the Autolab Esprit SPR user’s manual at 20°C. DrRecA (0.5 to 3 μM) was used for the mobile phase (20 mM Tris [pH 7.6] and 1 mM MgCl_2_). After the subtraction of mobile-phase buffer controls, the data were processed using Autolab kinetic evaluation software (V5.4) and plotted after curve smoothing using GraphPad Prism software.

### DNA strand exchange reaction.

Extended-homology-dependent RecA-dependent DNA strand exchange was carried out as described previously (36, 52). The DrRecA-dependent DNA strand exchange reaction was carried out at 37°C using M13mp18 circular ssDNA and linear dsDNA. The reaction was carried out in buffer (25 mM Tris-acetate, 1 mM DTT, 5% glycerol, 3 mM potassium glutamate, 10 mM magnesium acetate, and an ATP-regenerating system [10 U/mL of pyruvate kinase–3.3 mM phosphoenolpyruvate or 10 U/mL creatine kinase–12 mM phosphocreatine]). The DNA, SSB, ATP, DrRecA, and DrDprA protein concentrations are indicated for each experiment. The reaction was initiated by the preincubation of ssDNA with the DrRecA protein at 37°C for 5 min, followed by the addition of ATP and the SSB protein. After incubation for 5 min, linear duplex DNA was added to start the DNA strand exchange reactions. The DrDprA protein was added before the addition of dsDNA (wherever required). To check the inhibitory effect of SSB on the SER, SSB (0.3 to 1.2 μM) was added before the addition of DrRecA. The reactions were stopped by adding 5 μL of stop solution (0.125% bromophenol blue, 25 mM EDTA, 25% glycerol, 5% SDS, and 0.5 μg proteinase K) to the mixture, and the samples were incubated for another 10 min at 37°C. The samples were electrophoresed in a 0.8% agarose gel with Tris-acetate-EDTA (TAE) buffer. Gels were stained with ethidium bromide and photographed using a gel documentation system (Syngene).

For the oligonucleotide-based DNA strand exchange reaction, 0.5 μM DrRecA was incubated with a 167-mer (2.5 μM nucleotides) in 10 μL of buffer (25 mM Tris-HCl [pH 7.5], 1 mM DTT, 2.5 mM MgCl_2_, 25 mM KCl) containing 1 mM ATP for 5 min; after this, a ^32^P-labeled 40-mer dsDNA oligonucleotide (2.5 μM nucleotides) was added. The DrDprA protein was added when required at the indicated concentrations. To terminate reactions, stop solution (0.125% bromophenol blue, 25 mM EDTA, 25% glycerol, 5% SDS, and 0.5 μg proteinase K) was added to the mixture, and the samples were incubated at 37°C for 10 min. The samples were analyzed on a 10% PAGE gel, the dried gel was exposed to X-ray film, and an autoradiogram was developed.

### Cloning and site-directed mutagenesis.

D. radiodurans genomic DNA was prepared using Genomic DNA isolation kit, Board of Radiation & Isotope Technology (BRIT), DAE. The DrDprA coding sequence was PCR amplified using gene-specific primers (see Table S1 at https://barc.gov.in/publications/mbio/dna_mp/spectrum-03470-22.pdf) and cloned into the pBAD vector at the XhoI and EcoRI sites. The resultant plasmid, pBAD*dprA*, was used for site-directed mutagenesis (SDM) of DrDprA. The putative amino acids of DrDprA responsible for the DprA-RecA interaction (G253 and D277), the DprA-DprA interaction (L293 and H284), and the interaction with DNA (R137, R234, and F229) were selected, and SDM was performed according to the kit manufacturer’s protocols (New England BioLabs). *In vitro* mutagenesis was confirmed by sequencing. The resultant pBAD plasmids expressing G253R, D277A, L293A, H284A, R137D, R234D, and F229S mutants of DrDprA were named pBADG253R, pBADD277A, pBADL293A, pBADH284A, pBADR137D, pBADR234D, and pBADF229S, respectively. These plasmids were transformed into E. coli Top10 cells for the expression of recombinant proteins.

### DNA binding assay.

As described above, the DNA binding activities of DrDprA and its mutant derivatives were checked using an electrophoretic gel mobility shift assay (EMSA) (35). In brief, a 40-bp- or 167-bp-long random-sequence oligonucleotide (see Table S1 at https://barc.gov.in/publications/mbio/dna_mp/spectrum-03470-22.pdf) was used as the ssDNA substrate. The dsDNA substrate was made by annealing it with its complementary strand (see Table S1 at the URL mentioned above). Both ssDNA and dsDNA were labeled with [γ-^32^P]ATP using polynucleotide kinase and purified with a G-25 column. The 2 nM labeled probe (ssDNA and dsDNA) was incubated with increasing concentrations of DrDprA in a 10-μL reaction mixture containing 10 mM Tris-HCl (pH 7.5), 50 mM NaCl, and 1 mM DTT for 10 min at 37°C. The products were analyzed on a 10% native polyacrylamide gel and dried, and the signals were recorded by autoradiography. The DNA band intensity in the free form or bound to protein was quantified using ImageJ software. The bound fraction (percentage) of DNA was plotted against the protein concentration using GraphPad Prism. The *K_d_* for curve fitting of the individual plots was determined by the software working on the principle of the least-squares method, applying the formula *Y* = *B*_max_ × [*X*]/*K_d_* + [*X*], where [*X*] is the protein concentration and *Y* is the bound fraction. The fraction (percentage) of the DNA bound to protein was plotted as a function of the protein concentration using GraphPad Prism. To determine the DrDprA DNA binding preference between ssDNA and dsDNA, a competition assay was performed where the binding of 0.2 nM ssDNA or dsDNA was challenged with unlabeled homologous ssDNA or dsDNA (10 nM to 80 nM), respectively. The log *K_i_* value was calculated by curve fitting using nonlinear regression of the competition binding equation for the one-site-fit *K_i_* in GraphPad Prism software.

DrDprA binding with M13 DNA was carried out in a 10-μL reaction mixture containing 10 mM Tris-HCl (pH 7.5), 50 mM NaCl, and 1 mM DTT for 10 min at 37°C. The DNA concentration used was 1 μM, and the protein concentrations used were 1 and 2 μM. A total of 1 mM ATP was used where indicated.

### Biophysical characterization.

The quality of the purified recombinant proteins was assayed by circular dichroism (CD) spectroscopy. In brief, 0.5 μM protein was diluted in a buffer containing 20 mM Tris (pH 7.6) and 100 mM NaCl, and the spectra were recorded using a CD spectrophotometer (J815; JASCO, Japan).

The polymerization dynamics of purified recombinant proteins were monitored by dynamic light scattering (DLS) and size exclusion chromatography. In brief, the solutions/buffers used were filtered through a 0.22-μm filter, and the protein was centrifuged at 14,000 rpm for 60 min at 4°C before DLS and size exclusion chromatography were performed. The DLS spectra were recorded with 5 μM purified proteins in buffer (20 mM Tris [pH 8], 100 mM NaCl, and 0.1 mM EDTA). Data were recorded for 10 s at 37°C using the Anton Paar Litesizer 500 system. The volume-weighted relative frequency (percent) was plotted against the particle diameter (nanometers) using GraphPad Prism software. For size exclusion chromatography, a Superdex-200 Increase 10/300 GL column (GE) was used, and the absorbance (mAu) was plotted as a function of the elution volume (milliliters) using GraphPad Prism.
